# Uses of Traumaticine

**Published:** 1885-07

**Authors:** A. Lagorio

**Affiliations:** Chiavari, Italy


### USES OF TRAUMATICINE.

Dr. C. Costa calls attention in *La Salute* to the beneficial effects obtained by traumaticine in many cutaneous diseases, such as psoriasis, pityriasis versicolor, scrofuloderma and lupus erythematodes.

To Auspitz of Vienna belongs the credit of the introduction of this remedy and the indications for its uses. Traumaticine is a solution of one part of gutta-percha in ten parts of chloroform. It is a syrupy liquid, smells strongly of chloroform, and has a color and a consistence according to the quality of gutta-percha used; of this there are three qualities. The *brown* is that which is most abundantly found in commerce. It dissolves with greater difficulty. It dries more slowly, but preserves its elasticity longer. The *red* is that used by dentists.

It dissolves more readily in the proportion of 10 % and also of 20 % : it forms a liquid of a rosy color, and it is to be preferred where it is to be applied to exposed parts of the body, as the face, hands, etc. The *white* dissolves quickly in chloroform in the proportions of 10 and 20 %.

These solutions when applied to the skin easily penetrate in every fold and irregularity, and thereon dry with moderate rapidity. They form a thin adherent pellicle, white, rosy or brown according to the quality of the gutta-percha. The traumaticine folds and bends according to the movements of the skin, without breaking or detaching itself, and without causing any pain, compression or excessive stretching. Numerous remedies can be suspended in it according to their indications, as crysarobin at 10 % or more, as also sulphur, iodoform, salicylic acid, boric and pyrogallic acid and other subtances.

For the first time it has been used by him in the treatment of acute blennorrhagic epididymitis, applied in layers externally. Before applying it, the scrotum is to be shaved, and ice is to be applied to it for half an hour so as contract it and reduce its size. By this method the swelling has rest, protection and compression, and rapidly disappears.

Costa praises the applications of traumaticine as the best remedy in these disorders.

A, LAGORIO.

Chiavari, Italy, 27th May, 1885.

